# Security and Privacy in Wireless Sensor Networks: Advances and Challenges

**DOI:** 10.3390/s20030744

**Published:** 2020-01-29

**Authors:** Cheng-Chi Lee

**Affiliations:** 1 Department of Library and Information Science, Research and Development Center for Physical Education, Health, and Information Technology, Fu Jen Catholic University, New Taipei City 24205, Taiwan; cclee@mail.fju.edu.tw; 2 Department of Photonics and Communication Engineering, Asia University, Taichung 41354, Taiwan

## 1. Introduction

Wireless sensor networks (WSNs) have evolved over the last few decades due to the availability of low-cost, short-range and easy deployed sensors. WSN systems focus on sensing and transmitting the real-time sense information of a specific monitoring environment for the back-end system to do further processing and analysis. However, due to the publicity of wireless communication channels, security and privacy concerns with WSN systems have becoming a hot topic of discussion. This Special Issue aims to solicit the state-of-the-art research articles on advanced technologies for WSN systems, which encompass all types of research activity, such as the design, development, challenges and application of service models. 

In wireless communication channels, ensuring the security and privacy of the WSN will be one of the most important and critical issues. To protect communication data from being eavesdropped, altered, or forged by illegal nodes, it is generally taken to use encryption/decryption and a digital signature mechanism to solve the privacy issues. Another important issue is the issue of authentication. To protect the resources from being used by illegal nodes, authentication mechanisms are generally used to achieve this goal. However, under the circumstances that the battery capacity of the sensing node is generally limited, all of the protection mechanisms that we have developed hope to extend the use time of the sensing network—that is, the computational complexity cannot be too high.

In this Special Issue, we received 27 papers in total, and 12 of them were accepted and published. The authors have presented some novel ideas and methods to solve the problem of security and privacy in WSN. We believe the completely secure and efficient sensing environments can benefit humankind worldwide. We would like to thank all of the authors for their contributions to this field.

## 2. Summary of the Special Issue

Finding the right balance between required security and implementation capabilities makes WSN more challenging. The published papers presented their important contributions between required security and implementation capabilities. To achieve privacy, we generally use public-key cryptography algorithms or symmetric cryptography algorithms in WSN. It is the natural choice to use a symmetric algorithm due to its complexity. Reference [1] presents a generic model of the pseudo-random generator in computationally constrained environments. It can be applied to stream cryptography algorithms, as a subgroup of symmetric cryptography algorithms, in WSN and the Internet of Things (IoT). The proposed scheme is suitable for the implementation of the security solution in the computational constrained microprocessor environments, e.g., WSN and IoT.

In symmetric algorithms, we need a link key to encrypt or decrypt communicated messages between two nodes. The Secrecy Amplification (SA) protocol aims to improve the overall security of a network of interconnected nodes in the case that a non-trivial proportion of the link keys have become compromised. Reference [2] presents an efficient SA protocol for the Arduino and TinyOS platforms. The authors also verify some SA protocols by simulations in a real network. Their proposed SA protocol won in the end.

Recently, Wireless Visual Sensor Networks (WVSNs) have been widely used to encrypt images in many fields. Reference [3] presents an image encryption scheme based on compressive sensing and non-uniform quantization in WVSN. In the proposed scheme, an optimized logistic map is designed to expand the parameter value space and eliminate the period windows in current chaotic systems. Their results prove that the proposed scheme is better than that in existing schemes under the condition of strong noise interference or severe data loss for WVSNs.

Although it is the natural choice to use a symmetric algorithm to protect communication data in WSNs, the symmetric algorithms still have restrictions for WSNs. In some applications, we still need public-key cryptographic algorithms. The most famous of public-key cryptographic algorithms is RSA. Reference [4] implements 1024-bit RSA on a constrained microcontroller MSP430, which is a commonly used microcontroller in WSNs. To accelerate RSA operations, the authors utilized several acceleration techniques, such as the subtractive Karatsuba–Ofman, Montgomery multiplication, operand scanning, Chinese remainder theorem and sliding window methods. Their implementations achieved better timings than the existing works.

Reference [5] presents a framework for contrasting a secure domain of sensor nodes. The authors wanted to integrate some security measures to build a secure environment in a WSN. They presented several procedures which were prepared to cover all stages of the life of the entire secure domain. The proposed solution ensures the authentication of sensor nodes and their resistance against unauthorized impact with the hardware/software configuration. From their experimental results, the proposed framework is secure and practical.

The cloud-assisted WSN provides a promising solution to handling massive data. How to efficiently access the encrypted and decrypted massive data in a cloud-assisted WSN is a key challenge. Reference [6] presents a secure and efficient data sharing and searching scheme in a WSN. The proposed scheme is secure against both off-line and on-line keyword guessing attack performed by external and internal adversaries. Their results prove that it can achieve keyword security and document security. Furthermore, the proposed scheme is more efficient than previous schemes.

Reference [7] presents a vulnerability assessment of sensor systems. The authors develop a new Common Criteria-compliant method to specify the vulnerability assessment process and related data in a structured way for WSNs. They also show that their validation on a sensor example. The research results will be used as input for the National project “National schema for the security and privacy evaluation and certification of IT products and systems compliant with Common Criteria”. 

Reference [8] presents a self-embedding authentication method that helps to detect and locate tampered areas as well as to recover the tampered area. They introduced two types of detection method: block-wise and pixel-wise methods. The methodology was validated using six grayscale images of size 512 × 512, which showed that it has a better performance for tamper detection and image recovery even in highly tampered images.

Reference [9] presents a model for the problem of privacy-preserving access control in IP-enabled WSNs, namely eHAPAC. They integrated Hidra access control and an APAC model to ensure privacy and unlinkability in resource-constrained devices and improved the group signature-based APAC model to prevent arbitration organizations from cheating. The results of the security analysis show that their model can help to increase the flexibility of public key management and resist resource consumption attacks.

In Reference [10], the authors targeted a fundamental issue, in that the security and efficiency of a routing model has to be guaranteed before we can enjoy the applications in a WSN. They presented a trusted routing scheme based on the blockchain and reinforcement learning to prevent malicious node attacks. This work reported that it still has a good delay and throughput performance even in the routing environment with 50% malicious nodes.

The intrusion detection (ID) is an important subject in the field of the security of WSNs. Reference [11] presented an ID method based on the synthetic minority oversampling technique (SMOTE) and random forest algorithm. The random forest algorithm combined with the SMOTE provided an effective solution to solve the problem of class imbalance and improves the classification accuracy of ID. In their simulation, the accuracy of ID of the proposed scheme is higher than previous schemes.

The wireless body area network (WBAN) is used to monitor patients’ real-time health status and seamlessly transmit physiological data to medical institutions including hospitals, community clinics and emergency centers. Reference [12] presents a secure and efficient group key management protocol with cooperative sensor association in WBANs. The authors are the first to propose the system model providing a specific group communication channel for message broadcasting between healthcare center and patients. From their performance analysis, it was demonstrated that the proposed protocol is more secure and efficient than the other group key management protocols in WBAN.

## References

[1] Unkašević T., Banjac Z., Milosavljević M. (2019). A Generic Model of the Pseudo-Random Generator Based on Permutations Suitable for Security Solutions in Computationally-Constrained Environments. Sensors.

[2] Ostadal R., Matyas V., Svenda P., Nemec L. (2019). Crowdsourced Security Reconstitution for Wireless Sensor Networks: Secrecy Amplification. Sensors.

[3] Shen Q., Liu W., Lin Y., Zhu Y. (2019). Designing an Image Encryption Scheme Based on Compressive Sensing and Non-Uniform Quantization for Wireless Visual Sensor Networks. Sensors.

[4] Gulen U., Alkhodary A., Baktir S. (2019). Implementing RSA for Wireless Sensor Nodes. Sensors.

[5] Furtak J., Zieliński Z., Chudzikiewicz J. (2019). A Framework for Constructing a Secure Domain of Sensor Nodes. Sensors.

[6] Zhu B., Susilo W., Qin J., Guo F., Zhao Z., Ma J. (2019). A Secure and Efficient Data Sharing and Searching Scheme in Wireless Sensor Networks. Sensors.

[7] Bialas A. (2019). Vulnerability Assessment of Sensor Systems. Sensors.

[8] Lee C.-F., Shen J.-J., Chen Z.-R., Agrawal S. (2019). Self-Embedding Authentication Watermarking with Effective Tampered Location Detection and High-Quality Image Recovery. Sensors.

[9] Liu F., Tang Y., Wang L. (2019). eHAPAC: A Privacy-Supported Access Control Model for IP-Enabled Wireless Sensor Networks. Sensors.

[10] Yang J., He S., Xu Y., Chen L., Ren J. (2019). A Trusted Routing Scheme Using Blockchain and Reinforcement Learning for Wireless Sensor Networks. Sensors.

[11] Tan X., Su S., Huang Z., Guo X., Zuo Z., Sun X., Li L. (2019). Wireless Sensor Networks Intrusion Detection Based on SMOTE and the Random Forest Algorithm. Sensors.

[12] Tan H., Chung I. (2018). A Secure and Efficient Group Key Management Protocol with Cooperative Sensor Association in WBANs. Sensors.

